# Identifying the psychosocial predictors of ultraviolet exposure to the face in patients with xeroderma pigmentosum: a study of the behavioural factors affecting clinical outcomes in this genetic disease

**DOI:** 10.1136/jmedgenet-2021-108323

**Published:** 2022-04-07

**Authors:** Robert Sarkany, Sam Norton, Martha Canfield, Myfanwy Morgan, Lesley Foster, Kirby Sainsbury, Vera Araujo-Soares, Hans Christian Wulf, John Weinman, Jessica Walburn

**Affiliations:** 1 Xeroderma Pigmentosum Unit, Guy's and St Thomas' NHS Foundation Trust, London, UK; 2 Health Psychology Section, Institute of Psychiatry, Psychology & Neuroscience, King's College London, London, UK; 3 School of Cancer and Pharmaceutical Sciences, King's College London, London, UK; 4 Population Health Institute, Faculty of Medical Sciences, Newcastle University, Newcastle upon Tyne, UK; 5 Health Technology and Services Research, Technical Medical Centre, University of Twente, Enschede, The Netherlands; 6 Department of Dermatology, Bispebjerg Hospital, Kobenhavn, Denmark

**Keywords:** dermatology, DNA repair, DNA damage, disorders of environmental origin

## Abstract

**Background:**

For patients with xeroderma pigmentosum (XP), the main means of preventing skin and eye cancers is extreme protection against ultraviolet radiation (UVR), particularly for the face. We have recently developed a methodology for objectively measuring photoprotection behaviour (‘UVR dose to facial skin’) and have found that the degree of photoprotection varies greatly between patients with XP. We have previously identified factors affecting photoprotection behaviour in XP using a subjective measure of photoprotection. Here, we have used this objective methodology to identify the factors which determine photoprotection behaviour in XP.

**Methods:**

We studied 29 psychological, social, demographic and clinical variables in 36 patients with XP. We have previously objectively measured UVR protection (by measuring the dose of UVR reaching the skin of the face over a 3-week period) in these patients. Here, we use linear mixed-effects model analysis to identify the factors which lead to the differences in degree of photoprotection observed in these patients.

**Results:**

Psychosocial factors accounted for as much of the interindividual variation in photoprotection behaviour (29%) as demographic and clinical factors (24%). Psychosocial factors significantly associated with worse UVR protection included: automaticity of the behaviours, and a group of beliefs and perceptions about XP and photoprotection known to associate with poor treatment adherence in other diseases.

**Conclusions:**

We have identified factors contributing to poor photoprotection in XP. Identifying these potentially reversible psychosocial features has enabled us to design an intervention to improve photoprotection in patients with XP, aiming to prevent skin and eye cancers in these patients.

**Key messages**

**What is already known on this topic**

- Ultraviolet radiation photoprotection is critical to prognosis in xeroderma pigmentosum (XP).
- We have recently found that many patients with XP photoprotect poorly.

**What this study adds**

- Out of 29 factors examined, 9 are strongly associated with poor photoprotection in patients with XP.
- Seven of these nine factors are psychological or social.

**How this study might affect research, practice and/or policy**

- Since these seven psychosocial factors are potentially reversible, we are designing a psychological intervention targetting these factors, in order to improve photoprotection in patients with XP.

## Introduction

Xeroderma pigmentosum (XP) is an autosomal recessive inherited disorder in which patients develop multiple skin cancers from childhood, eye disease (corneal and conjunctival scarring and malignancy) and progressive neurological degeneration.^1^ The incidence in Western Europe is low (2.3 per million live births).^2^ XP is more common in Japan^3^ and

North Africa.^4^ Mean life expectancy in the USA is 32 years.^5^ The prognosis in tropical countries is much worse: in South Africa 80% of patients develop multiple squamous cell carcinomas, frequently metastatic, by the age of 6.^6^ In 80% of patients, the disease is caused by defects in nucleotide excision repair (NER), required for repair of ultraviolet radiation (UVR)-induced mutagenic photo-products in nuclear DNA in cells.^7^ The other 20% of patients are ‘XP variants’, who have normal NER but defective translesion synthesis past UVR-induced DNA damage.^7^ The genetic defects can involve any of eight disease-causing genes. Each gene corresponds to one XP complementation group (XP-A to G, and XP-V). There is clinical heterogeneity between and within complementation groups.^1^

In XP, disease phenotype correlates to some extent with the mutations,^1^ but, because the molecular defect specifically impairs the cellular response to DNA damage caused by UVR, UVR exposure of the skin and eyes plays the critical role in determining clinical outcomes.

Since there is no therapy for the underlying molecular defect, the main means of preventing eye and skin cancers to minimise UVR exposure. This is achieved through absolute and lifelong photoprotection. Since 5% of daylight, even when cloudy, is UVR, patients require rigorous avoidance of daylight, protection of the face using visors made of UVR-protective transparent film and of the rest of the body with clothing, hats, gloves and high factor sunscreens.^8^ The advice for photoprotection is considerably more rigorous in XP than is recommended for other photosensitive conditions or for other groups at increased risk of skin cancer. Since 80% of skin cancers in XP are on the face, head and neck,^9^ and skin cancers on the face have the highest surgical morbidity, photoprotection of the face is particularly important in XP.

If there are any patients with XP who photoprotect poorly, it is likely that this will significantly worsen their prognosis. Over the past 20 years, it has become clear that patients’ ‘non-adherence’ to medical treatments contributes to worse clinical outcomes in many diseases.^10^ It is estimated that 30%–50% of all prescribed treatments are not taken as directed^11^ and that rates might be higher in dermatological conditions^12 13^ and for preventive health behaviours.^14^ There are many known determinants of non-adherence (capability (‘is the patient able to adhere to the treatment?’), opportunity (‘is the treatment available and affordable for the patient?’) and motivational (‘does the patient wish to adhere to the treatment?’).^15^ Out of all these factors, a small group of psychological and social factors, including perceived necessity and concerns about treatment, the extent to which adherence becomes habitual and patients’ mood and level of confidence to enact the behaviour, are consistent predictors of non-adherence in a range of chronic diseases.^16–18^ Since these factors can be modified by behaviour change interventions,^19^ identifying factors associated with poor photoprotection has therapeutic implications in XP.

Prior to our current programme of research, there had been no studies of how well patients with XP photoprotect, or of the factors determining their photoprotection behaviour.

In our previous work, we have used a simple self-report questionnaire to subjectively assess UVR protection for the face in 156 patients with XP in Europe and the USA.^20^ The results suggested that UVR protection for the face was suboptimal in one-third of these patients. That questionnaire study of XP^20^ also identified several psychosocial factors which appeared to be associated with poor adherence to photoprotection. However, the significance of the results from that study is unclear because the measure of adherence to photoprotection was so subjective.

In the study presented in this paper, we identify the psychosocial factors associated with poor photoprotection, using the ‘gold standard’ of UVR protection in XP, an objective and quantitative measure of the dose of UVR to which the patient’s face is exposed.

Since there has previously not been a way to objectively measure the dose of UVR reaching the face, we recently developed a method to do this.^21 22^ It measures the total dose of UVR reaching the skin of the face per day, taking the methods of face photoprotection used by the patient into account. For each 15 min period a patient spends outside (window glass efficiently protects against UVR when inside), we measure the environmental UVR (using a wrist-worn UVR dosimeter), and combine that with the proportion of environmental UVR which will penetrate the method of face photoprotection being used during that period (using the ‘face protection factor’ associated with the protection method recorded by the patient in an activity diary for that period).^21 22^ We have used this method to objectively measure photoprotection behaviour in 36 patients with XP in the UK over a 21-day period.^22^ We identified a wide range in photoprotection behaviour: the patient with XP with the highest mean daily UVR exposure dose to the face had 120-fold higher exposure than the patient with the lowest. The worst protecting patients had UVR exposure similar to the mean in a group of healthy individuals.^22^

In summary, our recent study has shown that poor adherence to photoprotection, assessed using this ‘gold standard’ objective measure, is a common problem in XP.^22^ In this current study, we have gone further in this same group of 36 patients, to examine their psychosocial, and other, characteristics in detail, in order to identify the factors which may explain why photoprotection is so unexpectedly poor in so many of them.

## Methods

In this prospective observational study, patients completed a structured questionnaire to measure psychological and social variables. Clinical and demographic variables were collected from patients’ medical records. The objective measure of photoprotection was the mean daily dose of UVR to which the skin of the face was exposed over the 3-week period of the study.

### Recruitment

Patients were recruited from the UK National XP Clinic. The inclusion criterion was a diagnosis of XP made by identifying reduced unscheduled DNA repair or typical XP-variant changes in cultured skin fibroblasts^23 24^ in a patient with typical clinical features,^1^ confirmed by genetic testing (mutations analysed in genomic DNA by massively parallel Illumina sequencing, using a platform containing the coding exons and splice sites (−30/+20 bp) of the 8 XP genes^1^).

Eligible patients were contacted by a research nurse. For patients under 16 years, and for adults lacking capacity to consent (due to XP-related cognitive impairment), their carer was contacted. Patients, or carers, were only recruited if they understood sufficient English to complete questionnaires and activity diaries.

### Procedure

#### UVR measurement and activity diary

Each patient wore a UVR electronic dosimeter (SunSaver 3^25 26^) on the wrist, and completed a simple one page per day UVR protection record in which the patient selected which method or methods of face photoprotection, from a list of 7 possibilities, they used for each 15 min period spent outdoors (or whether they had not protected at all).^22^ They did this throughout a 3-week period in 2016, during the months when environmental UVR levels are highest in the UK (6 May–6 August). All patients were provided with their usual SPF50 high UVA protection sunscreen to use for the duration of the study, and were trained to apply it by an XP clinic nurse. For each 15 min period, the dose of UVR to the face was calculated by multiplying the UVR exposure recorded by the wrist-worn dosimeter by the ‘face protection factor’ associated with the face photoprotection behaviour recorded in the diary for that interval.^22^ The UVR measurements from the dosimeter were expressed as ‘standard erythemal doses’ (SEDs), a measure chosen because it is weighted towards the wavelengths of UVR most damaging to DNA, that is, the SED is the clinically relevant measure of UVR exposure in XP.^22 27 28^ Details of the UVR face exposure measurement methodology are described elsewhere.^21 22^

#### Demographic and clinical factors

Clinical and physical variables collected from patients’ medical records from the XP clinic included: XP complementation group, genotype, cultured skin fibroblast unscheduled DNA repair activity, presence/absence of XP-related cognitive impairment, severity of eye and neurological disease, degree of photosensitivity (‘XP Sunburn score’^29^), number and type of skin and eye cancers, age at diagnosis, years since diagnosis, age when photoprotection started. Demographic data included age, gender, ethnicity and family history of XP.

#### Psychological and social factors: assessed from a self-report questionnaire

The self-report questionnaire assessed psychological and social factors which might be determinants of photoprotection (see online supplemental materials). The self-report questionnaire assessed psychological and social factors which might be determinants of photoprotection. Self-caring adults completed the questionnaire themselves. For non-self-caring adults, and for children (below 18 years), the questionnaire was completed by the main carer, since UVR exposure of non-self-caring adults and children is largely decided by the main carer. The questionnaire was the same in both cases except for use of personal pronouns, with the carer providing answers about the patient’s XP and the carer’s beliefs and perceptions.

10.1136/jmedgenet-2021-108323.supp1 Supplementary data

Decisions about which psychosocial factors to include were based on the psychosocial factors already known to affect treatment adherence in other diseases,^14^ the factors already known to affect photoprotection behaviour in healthy individuals^30^ and on psychological theories relevant to treatment adherence^31 32^:

- Patients’ perceptions of XP (‘consequences’, ‘timeline’, ‘personal control of XP’, ‘photoprotection control of XP’, ‘treatment control’, ‘identity’, ‘negative emotional representation’ and ‘perceived understanding’) were examined using single items from the Adapted Brief Illness Perception Questionnaire,^33^ scored on an 11-point scale (0–10), higher scores representing a stronger and more negative perception.
- Beliefs relating to the patient’s perception of the need for photoprotection were examined with an adapted version of the Beliefs about Medicines Questionnaire.^34^ Participants indicated their strength of agreement with each statement on a 5-point scale from 1 (‘strongly disagree’) to 5 (‘strongly agree’), and a mean score for the two subscales (‘necessity’ and ‘concern’) is calculated, higher scores indicating stronger beliefs.
- ‘Intention’, ‘self-efficacy’ and ‘automaticity’ for each photoprotection behaviour when outside (eg, wearing a face visor, using sunscreen, wearing a hat) were assessed by recording the strength of agreement with statements on a 7-point scale. The 10-item ‘intention’ and ‘self-efficacy’ scales were adapted from a manual based on the Theory of Planned Behaviour,^35^ and the 10-item automaticity scale was adapted from the Self-Report Habit Index.^36^ The mean score across items in each scale was calculated, higher scores indicating stronger agreement. A single item about the habit of avoiding going outside during the day was assessed with the same 7-point scale.
- The level of social support received (1 (‘no support’) to 5 (‘comprehensive support’)) and satisfaction with social support (1 (‘very dissatisfied’) to 5 (‘very satisfied’)) were adapted from the Social Support Questionnaire.^37^
- Emotional well-being was measured using the short-form Warwick-Edinburgh Mental Well-Being Scale (SWEMWBS).^38^ It consists of seven items measuring how often in the past 7 days participants experienced various positive aspects of mental well-being, each item scored on a 5-point scale (1 (‘none of the time') to 5 (‘all of the time’)). Total scores range from 7 to 35, higher scores indicating greater mental well-being.

We have previously established the reliability of these psychological scales in patients with XP: Cronbach’s α internal reliability values within a sample of 156 patients with XP were good: 0.73 for necessity, 0.80 for concern, 0.73 for intention, for 0.75 self-efficacy and 0.71 for automaticity.^20^

### Analysis

Descriptive information for continuous variables was reported as mean and SD, unless data were heavily skewed in which case the median and IQR was considered more appropriate. Categorical variables were reported as the count of non-missing observations and the percentage.

Factors associated with mean daily UVR exposure to the face were assessed using linear mixed-effects models. Daily total UVR exposure to the face was the outcome variable (ie, up to 21 repeated daily values per person). The model incorporated a random intercept to account for the repeated daily observations within individuals. A first-order autoregressive error structure was modelled to account for cross-day effects within individuals. Separate models were estimated for each predictor to assess the individual association of each predictor with UVR dose to the face. A multivariate model included all predictors identified in those models to estimate the overall variance in UVR dose to the face explained by clinical, demographic and psychosocial factors. Models controlled for weekend effects, whether the respondent rated the day as sunny, and the type of report (patient or carer). All analyses were conducted in Stata V.16 and the 5% level used to determine statistical significance.

### Exploratory analysis of qualitative groupings

We also carried out an exploratory analysis of the relationship between the data from this study and qualitative findings from a previous study. Twenty of the 21 adults in this study had previously taken part in a qualitative study in which semi-structured interviews with patients were analysed using a framework approach.^39^ That study identified three psychologically distinct groups within the XP population: a ‘dominated’ group, who were very worried about the risks of XP and described photoprotection dominating their lives to a distressing extent; a ‘resistant’ group, for whom photoprotection was not important, and whose priorities were to avoid stigma, be accepted and engage in normal social activities and an ‘integrated’ group, for whom photoprotection was a routine and accepted part of their life.

Here, we have analysed the relationship between the results from this study (mean daily UVR dose to the face, and psychosocial variables from the questionnaire) and the group (‘dominated’, ’resistant’ or ‘integrated’) to which each patient was found to belong in the previous qualitative study. Patient numbers in the three subgroups were too low to enable rigorous statistical analysis.

## Results

Of the 93 patients with XP known to the XP clinic, 78 were eligible, 47 of whom consented to take part. Six withdrew before dosimeter fitting, one provided <14 days of data, in two dosimeters malfunctioned and two did not provide a full analysable dataset. In total, 36 of the 47 patients providing consent provided sufficient data to be included in the analysis. This occurred despite the demands on patients of the study. Because of the rarity of the disease, the sample size was based on the maximum number of patients that could be recruited, rather than on a calculation of statistical power.

### Baseline characteristics

*Demographic and clinical characteristics* (table 1): the age range was wide (mean age 29.2, SD=18.8). Most patients (21 of the 36) were self-caring adults, with a wide range of educational attainment. Of the 15 non-self-caring patients, 11 were children, and 4 were cognitively impaired adults. There were more male patients than female patients. Seventeen of the patients were white British and 19 were non-white British (15 British Asian (13 Pakistani, 1 Sri Lankan, 1 Indian), 2 mixed Caribbean ancestry, 1 Arab and 1 Turkish), with a similar ethnic breakdown in the self-caring adult and cared-for groups. All XP complementation groups were included apart from B, which is very rare. Cognitive impairment and eye problems due to XP were common. XP sunburn severity scores show that 44% of patients described abnormally severe sunburn reactions, as expected given the known links between sunburn responses and complementation group (only groups C, E and V have normal sunburning responses^34^). Around two-thirds of patients had started photoprotection by the age of 13 (mean 12.6, SD=13.2). Nearly 40% had already suffered a mucocutaneous malignancy, which had occurred at a wide range of ages, reflecting the clinical heterogeneity of this disease.^1^

**Table 1:** Demographic and clinical characteristics of the patients

	Self-caring adult Sample (n=21)	Patients cared for by a caregiver (children; non-self-caring cognitively impaired adults) Sample (n=15)	Total (n=36)
***Demographic variables***			
Male, n (%)	14 (67%)	9 (60%)	23 (64%)
Female, n (%)	7 (33%)	6 (40%)	13 (36%)
Age, mean (SD)	40.0 (16.0)	14.1 (9.9)	29.2 (18.8)
Ethnicity			
White British	9 (43%)	8 (53%)	17 (47%)
Asian British	8 (38%)	7 (47%)	15 (42%)
‘Other mixed’: mixed Caribbean	2 (9%)	0 (0%)	2 (5%)
‘Other’: Turkish	1 (5%)	0 (0%)	1 (3%)
‘Other’: Arab	1 (5%)	0 (0%)	1 (3%)
***Clinical variables***			
Complementation group			
A	5 (24%)	3 (20%)	8 (22%)
C	6 (29%)	5 (33%)	11 (31%)
D	1 (5%)	4 (27%)	5 (14%)
E	2 (10%)	0 (0%)	2 (6%)
F	1 (5%)	0 (0%)	1 (3%)
G	0 (0%)	2 (13%)	2 (6%)
V	6 (28.6%)	0 (0%)	6 (17%)
Unknown	0 (0%)	1 (7%)	1 (3%)
Age at diagnosis (years), mean (SD)	20.4 (15.9)	4.2 (3.4)	13.7 (14.7)
Age at which started photoprotection (years), mean (SD)	18.9 (14.0)	3.7 (3.2)	12.6 (13.2)
Abnormal sunburn reaction (XP sunburn severity score ≥1), n (%)^30^*	6 (29%)	10 (67%)	16 (44%)
Previous skin, eye or oral malignancy, n (%)	12 (57%)	2 (13%)	14 (39%)
Cognitive impairment, n (%)	2 (10%)	5 (33%)	7 (19%)

*Patients with complementation groups A, B, D, F and G have abnormal, increased sunburn reactions. Groups C, E and V are associated with normal visible reactions to sun exposure.

XP, xeroderma pigmentosum.

*Psychological and social characteristics (table 2):* overall, participants perceived XP to be a chronic condition that can be controlled by photoprotection and medical procedures. They believed photoprotection to be necessary, but had moderate concerns about protecting. Respondents reported moderate intention to photoprotect (mean score=4.7, SD=1), rated photoprotection as fairly automatic (ie, happened without thinking: mean score=4.4, SD=1.6) and were confident that they were able to carry out each photoprotection activity (mean score=6.4, SD=1.3). They were less confident that they could avoid going outdoors (mean score=4.2, SD=2). Overall, the sample had a similar level of emotional well-being (mean score=23.0, SD=5.5) to the general population in England (mean score=23.6, SD=3.9)^40^ and felt that they were well supported (mean score=4.1, SD=1.1). In comparison to the self-caring adults, the cared-for sample (n=15) thought XP had more serious consequences (cared-for sample mean score=9.2, SD=1.4; self-caring adults’ mean score=6.0, SD=3.3), felt that they had less control (cared-for sample mean score=4.1, SD=4.1; self-caring adults’ mean score=6.1, SD=2.6) and thought that they had a poorer understanding of XP (cared-for sample mean score=6.3, SD=3.7; self-caring adults’ mean score=8.5, SD=1.4).

**Table 2:** Psychological and social characteristics of the patients

Psychological and social variables, mean score (SD)	Self-caring adult Sample (n=21)	Patients cared for by a caregiver (children; non-self-caring cognitively impaired adults) Sample (n=15)	Total (n=36)
*Illness perceptions (0–10)*			
‘Consequences’ of XP on life (0 no effect; 10 severe effect)	6.0 (3.3)	9.2 (1.4)	7.3 (3.1)
Timeline or duration of XP (0 a very short time; 10 forever)	9.4 (2.2)	9.7 (1.3)	9.5 (1.9)
Personal control of XP (0 absolutely no control; 10 extreme amount of control)	6.1 (2.6)	4.1 (4.1)	5.3 (3.4)
Photoprotection control of XP (0 not at all helpful; 10 extremely helpful)	8.0 (2.5)	9.8 (0.6)	8.8 (2.1)
Treatment control of XP (0 not at all helpful; 10 extremely helpful)	8.8 (1.8)	9.2 (2.1)	8.9 (1.9)
‘Identity’, that is, symptom experience (0 no symptoms at all; 10 many severe symptoms)	5.7 (2.9)	7.0 (3.4)	6.2 (3.1)
Illness concern (0 not at all concerned; 10 extremely concerned)	6.6 (2.9)	7.5 (2.8)	7.0 (2.8)
Understanding of XP (0 do not understand at all; 10 understand very clearly)	8.5 (1.4)	6.3 (3.7)	7.6 (2.8)
Emotional representation/impact of XP (0 not at all affected emotionally; 10 extremely affected emotionally)	5.0 (3.5)	7.0 (3.5)	5.9 (3.6)
*Beliefs about photoprotection (1 strongly disagree; 5 strongly agree)*			
Necessity (importance of UVR protection to the person’s health)	3.9 (0.8)	4.6 (0.8)	4.2 (0.8)
Concern (concerns about negative impacts of UVR protection to the person’s health)	2.9 (1.1)	3.7 (0.6)	3.2 (1.0)
*Intention to photoprotect (1 strongly disagree; 7 strongly agree)*			
Intention to avoid going out in the next 7 days	4.4 (2.4)	4.5 (2.4)	4.5 (2.4)
Intention to photoprotect outdoors (a variety of methods of photoprotection) in the next 7 days	4.5 (0.9)	5.2 (0.9)	4.7 (1.0)
*Confidence about ability to photoprotect (1 strongly disagree; 7 strongly agree)*			
Self-efficacy to photoprotect (Over the next 7 days I am *confident* I could … (a variety of methods of photoprotection)	6.2 (1.4)	6.6 (1.1)	6.4 (1.3)
Self-efficacy to avoid going outside	4.2 (2.0)	4.1 (2.5)	4.2 (2.2)
*Degree to which photoprotection is enacted automatically (1 strongly disagree; 7 strongly agree)*			
Automaticity of photoprotection (a variety of methods of photoprotection)	3.8 (1.5)	5.3 (1.2)	4.4 (1.6)
Automaticity to avoid going outside	3.7 (2.9)	4.9 (2.9)	4.2 (2.9)
*Social support (1 no support/very dissatisfied; 5 comprehensive support/very satisfied)*			
Level of social support	3.9 (0.9)	3.8 (1.3)	3.8 (1.0)
Satisfaction with support	4.1 (1.0)	4.0 (1.3)	4.1 (1.1)
*Psychological well-being (SWEMWBS) (7–35*)	22.9 (5.8)	23.0 (4.6)	23.0 (5.3)

SWEMWBS, short-form Warwick-Edinburgh Mental Well-Being Scale; UVR, ultraviolet radiation; XP, xeroderma pigmentosum.

### Associations between demographic, clinical and psychosocial factors and mean daily UVR dose to the face

**Figure 1:**
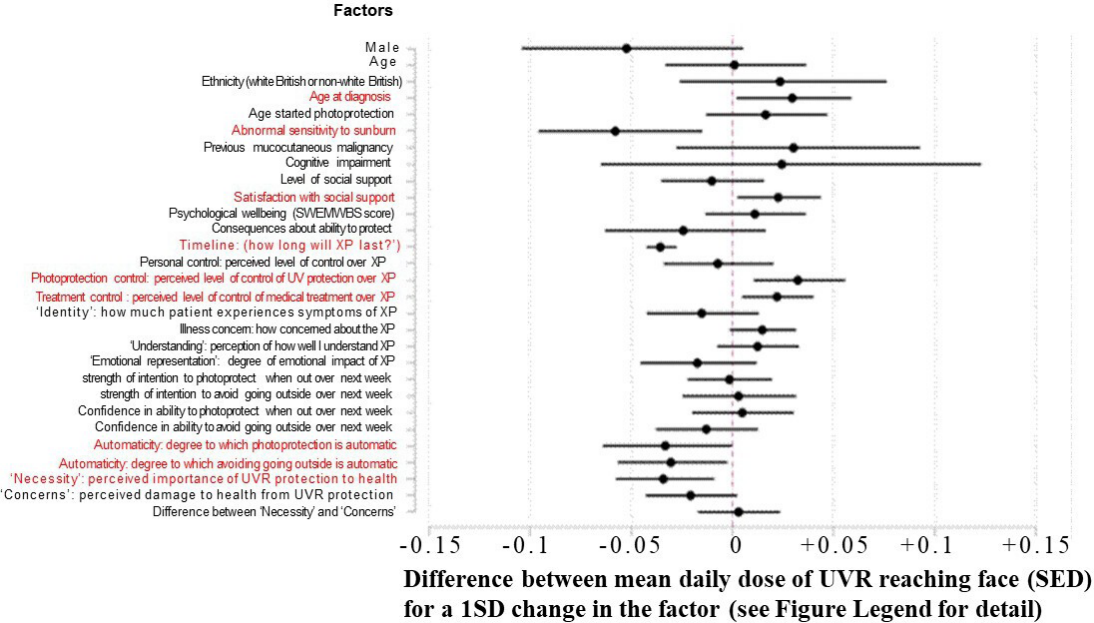
The associations between demographic, clinical and psychosocial factors and the calculated mean daily dose reaching the face (standard erythemal dose (SED)) for the patients with xeroderma pigmentosum (XP). The bars represent 95% CIs for the difference between the mean daily dose of ultraviolet radiation (UVR) reaching the face (SED) for a 1 SD change in the predictor variable for continuous variables, and change relative to reference group (eg, ‘males’ vs ‘females’) for categorical variables. The factors on the figure which can be seen to have a significant association with mean daily dose of UVR reaching the face are highlighted in red for clarity. SWEMWBS, short-form Warwick-Edinburgh Mental Well-Being Scale.

Out of the 29 demographic, clinical and psychosocial factors examined, 9 had statistically significant associations with photoprotection behaviour: 2 of them were clinical factors and 7 were psychosocial (figure 1, table 3).

**Table 3:** The associations between demographic, clinical and psychosocial factors and the calculated mean daily dose reaching the face (SED) for the patients with XP

Variable	Difference in mean daily UV dose to face for 1 SD change in predictor variable (SED)	95% CI		P value
		Lower	Upper	
**Timeline (how long will XP last?**)	−0.036	−0.042	−0.028	**0**
**Photoprotection control: perceived level of control of UV protection over XP**	0.032	0.011	0.056	**0.004**
**Abnormal sensitivity to sunburn**	−0.058	−0.096	−0.015	**0.008**
‘**Necessity’: perceived importance of UVR protection to health**	−0.034	−0.058	−0.009	**0.008**
**Treatment control: perceived level of control of medical treatment over XP**	0.022	0.005	0.04	**0.012**
**Satisfaction with social support**	0.023	0.002	0.044	**0.028**
**Automaticity: degree to which avoiding going outside is automatic**	−0.03	−0.057	−0.002	**0.033**
**Age at diagnosis**	0.03	0.002	0.059	**0.036**
**Automaticity: degree to which photoprotection is automatic**	−0.033	−0.064	0	**0.048**
Illness concern: how concerned about the XP	0.015	−0.001	0.031	**0.074**
Male	−0.052	−0.104	0.005	**0.075**
‘Concerns’: perceived damage to health from UVR protection	−0.021	−0.043	0.002	**0.079**
Understanding: perception of how well I understand XP	0.012	−0.007	0.033	**0.222**
Consequences of XP on life	−0.024	−0.063	0.016	**0.241**
Emotional representation: degree of emotional impact of XP	−0.017	−0.046	0.012	**0.246**
Age started photoprotection	0.016	−0.013	0.047	**0.275**
‘Identity’: how much patient experiences symptoms of XP	−0.015	−0.042	0.013	**0.289**
Previous mucocutaneous malignancy	0.03	−0.028	0.093	**0.311**
Confidence in ability to avoid going outside over next week	−0.013	−0.038	0.013	**0.32**
Ethnicity (white British or non-white British)	0.024	−0.026	0.076	**0.355**
Psychological well-being (SWEMWBS score)	0.011	−0.013	0.036	**0.376**
Level of social support	−0.01	−0.035	0.016	**0.438**
Cognitive impairment	0.024	−0.065	0.123	**0.602**
Personal control: perceived level of control over XP	−0.007	−0.034	0.02	**0.605**
Confidence in ability to photoprotect when out over next week	0.005	−0.02	0.03	**0.704**
Difference between ‘necessity’ and ‘concerns’	0.003	−0.017	0.023	**0.769**
Strength of intention to avoid going outside over next week	0.003	−0.025	0.031	**0.833**
Strength of intention to photoprotect when out over next week	−0.002	−0.022	0.02	**0.887**
Age	0.001	−0.033	0.036	**0.956**

This is the numerical data represented in figure 1. For clarity, the factors are presented in order of increasing p value, and those which can be seen to have an association with mean daily dose of UVR reaching the face, significant at the 5% level, are highlighted in red.

*Difference between the mean daily dose of UVR reaching the face (SED) for a 1 SD change in the predictor variable for continuous variables and change relative to reference group (eg, ‘males’ vs ‘females’) for categorical variables.

SED, standard erythemal dose; SWEMWBS, short-form Warwick-Edinburgh Mental Well-Being Scale; UVR, ultraviolet radiation; XP, xeroderma pigmentosum.

The clinical factors associated with worse photoprotection (ie, higher face UVR exposure) were as follows:

- Being older when diagnosed.
- Experiencing a normal sunburn response (ie, an abnormal severe sunburn response was associated with better photoprotection).

The psychosocial factors associated with worse photoprotection were as follows:

- Holding a strong belief that photoprotection can be beneficial for health (’photoprotection control’).
- Holding a strong belief that clinical treatment can control XP (‘treatment control’).
- Greater satisfaction with social support received for photoprotection,

The psychosocial factors found to be protective, that is, associated with a lower UVR dose to the face (figure 1 (the numbers represented in figure 1 are in table 3)) were:

- perceiving XP to be a life-long condition (‘timeline’);
- acceptance that photoprotection is necessary (‘necessity’);
- carrying out photoprotection automatically (‘automaticity’);
- avoiding going outdoors automatically (‘automaticity’).

A strong belief that XP has serious consequences (‘consequences’) was associated with better photoprotection but did not quite reach statistical significance.

Worse photoprotection was associated with better psychological well-being, although not reaching statistical significance.

None of the 20 other demographic, clinical or psychosocial factors analysed reached the 5% level of statistical significance for association with the UVR dose to the face. These included: sex, age, ethnicity, previous mucocutaneous malignancy and psychological measures of confidence and intention to photoprotect.

Exploratory analysis indicated that the protective effect of an abnormal sunburn reaction depended on the severity of the reaction, which differs greatly between complementation groups^29^: the most severe photosensitivity (XP sunburn severity score of 3) was associated with mean UVR dose to the face of 0.07 SED/day, <50% of the mean dose (0.15 SED/day) in those with less severe or no photosensitivity (XP sunburn severity scores 0–2). Patients with the less severe sunburn responses (XP sunburn scores 1–2) had a similar mean UVR dose to the face as patients without abnormal sunburn responses (sunburn score 0): 0.15 vs 0.14 SED/day.

Multivariate analysis indicated that 53% of the variance in mean daily UVR dose to the face (SED) could be explained by the demographic, clinical and psychosocial factors measured in this study. Psychosocial factors accounted for a greater share of this variance (29%) than demographic and clinical variables (24%).

#### Analysis of qualitative groups

In the 20 of the 21 adults in this study who had previously taken part in our qualitative interview study,^39^ we carried out exploratory analysis of the relationship between their results from the qualitative study and the data from this study (table 4).

**Table 4:** UV dose to face, and mean scores for some key psychosocial factors divided according to grouping from the qualitative interview study

	‘Dominated’ group	‘Integrated’ group	‘Resistant’ group
Number of patients	4	10	6
**Mean daily UV dose to face (SED/day**)	**0.04**	**0.12**	**0.2**
*Psychosocial factor*	*Mean score for psychosocial factor*	*Mean score for psychosocial factor*	*Mean score for psychosocial factor*
‘Timeline or duration of XP’ (0 a very short time; 10 forever)	9.5	10	8.8
‘Necessity’ of photoprotection (score 1–7)	4.5	4.1	3.4
‘Automaticity’ of photoprotection (score 1–7)	4.7	3.4	2.8
‘Consequences’ of XP on life (0–10)	8.3	5.5	5.9
Level of social support (1–5)	4.0	4.1	3.1

SED, standard erythemal dose; UV, ultraviolet; XP, xeroderma pigmentosum.

Analysis of UVR dose to the face in relation to these psychologically distinct subgroups^39^ (n=20), indicated, as expected, that the ‘dominated’ group had the smallest mean UVR dose to the face (0.04 SED/day), the ‘integrated’ group had a threefold higher dose (0.12 SED/day) and the ‘resistant’ group had a fivefold higher dose (0.20 SED/day). The ‘dominated’ group scored highest on the two psychosocial factors identified by this study as most protective: acceptance of the necessity of photoprotection and automaticity of photoprotection, whereas the ‘resistant’ group scored lowest on these factors.

## Discussion

We recently used a quantitative and objective measure of photoprotection (‘the UVR dose to the face’) to show that adherence to photoprotection is unexpectedly poor in many patients with XP. In this study, we have identified the factors which determine how well patients with XP photoprotect. UVR protection, particularly of the face, is crucial to prognosis.

In this study, multivariate analysis showed that the psychosocial factors examined are at least as important as clinical and demographic factors in determining objectively measured face photoprotection behaviour. The key psychosocial factors we have identified here, including perception of disease chronicity, treatment necessity beliefs and automaticity, are similar to but not identical with those identified in our previous questionnaire study in 156 patients with XP across Europe and the USA, which had used a subjective and qualitative self-report measure of face photoprotection.^20^ These are also some of the key determinants of non-adherence identified more broadly in other chronic diseases.^16 41^

In this study, we found that a strong belief that XP can be controlled by photoprotection was associated with a higher dose of UVR to the face. We suspect that this apparently paradoxical finding may be because individuals holding this belief strongly may overestimate the effectiveness of photoprotection measures taken while outside and therefore go outside more. Sunscreen and most other methods of face photoprotection let through 20% or more of UVR, so any factor that increases confidence to spend time outside, even with good protection, will increase overall UVR exposure of the face. Similarly, having a strong belief that clinical treatment is effective (with clinicians monitoring and removing skin cancers before they metastasise) was associated with an increase in UVR dose to the face. These unintended consequences of patients developing overconfidence in medical interventions has been shown to cause similar problems in cardiac patients: patients with the highest confidence in the effectiveness of medical intervention are less likely to adopt a healthier lifestyle after cardiac surgery.^42^ This highlights the tension between patients’ and health professionals’ responsibilities for health outcomes.

Exploratory analysis of the results from this study in the 20 adult patients who had also previously taken part in our qualitative interview study found that group differences in clusters of psychosocial factors (as demonstrated by the three patterns of adaptation to photoprotection identified in that qualitative study) were associated with differences in UVR dose-to-face larger than those ascribed to any individual psychosocial factors studied. This is consistent with health psychology theories,^31 32^ which suggest that psychosocial factors are not independent of each other, and tend to cluster together.

The complexity of the relationship between photoprotection in XP, well-being and social support is indicated by the finding that greater satisfaction with social support is associated with higher UVR exposure of the face. The qualitative interview findings in these patients^39^ had suggested that greater satisfaction with social support might reflect a more active social life, and that this might increase the time spent outdoors, and that finding has been confirmed by our results.

The lack of an association between lower UVR exposure and improved psychological well-being is unexpected. In many chronic diseases, better adherence to treatment is associated with improved psychological well-being, including patients with malignant melanoma in whom reduced sunbathing is associated with improved well-being.^43^ We suspect that this reflects the high demands of photoprotection in XP: the qualitative study found that excellent photoprotection was associated with considerable emotional distress among the ‘dominated’ group.^39^ This implies that there is a trade-off between dermatological and psychological health. The high social and psychological costs of rigorous photoprotection point to the importance of finding ways to cope with issues of stigma and self-identity as part of behavioural interventions to increase adherence among patients with XP, since adherence to photoprotection marks patients with XP out as visibly different, often leading to adverse social and psychological impacts.^44^

Studies of rare diseases are frequently limited by a sample size that restricts design and analysis, and present challenges for recruitment.^45^ Despite recruiting nearly half the known cases of XP in the UK, caution is needed when interpreting the associations between factors and UVR exposure outcomes, as our sample was small for capturing statistical significances and limited the types of statistical tests that could be conducted. For qualitative subgroup analysis, the sample was too small for statistical testing. Caution is also needed when interpreting the average scores for the psychological and social factors as some variables were not normally distributed. In addition, while we have accounted for possible measurement confounders in the mixed-effect models, the CIs were large across factors. If tested in a larger sample, the strength and direction of the associations could potentially be influenced by the interaction between demographic and clinical factors and/or other confounders.

The group of patients in this study are representative of the overall XP population in the UK demographically and clinically, including the proportion of adults to children.^1^ The previously published questionnaire study of adherence to photoprotection in XP was an international study of 156 patients with XP in the UK, France, the USA and Germany.^20^ In that study, 10 factors were identified which significantly affected photoprotection behaviour. Four of those factors (age at diagnosis, ‘necessity’ and both types of automaticity) make up four of the nine factors identified in this UK-based study with its stronger methodology. This suggests that the results of this UK-based study may have application to groups of patients outside the UK.

Although disease phenotype in XP correlates to some extent with the nature of the mutations,^1^ the eventual phenotype and clinical outcomes result from the interaction of the underlying genetic defect with acquired and environmental factors. Because the molecular defect in XP specifically impairs the cellular response to DNA damage caused by UVR, the environmental factor affecting disease expression is unusually well-defined. Exposure of the skin and eyes to environmental UVR, and protection from it, is entirely dependent on behaviour. An understanding of photoprotection behaviour and its underlying psychosocial causes provides an understanding of the acquired factor modulating phenotype in XP, to complement the understanding of the genetic factors. This study has defined the psychosocial basis of photoprotection behaviour in XP.

We have used the findings from this study to design a therapeutic behaviour change intervention (‘XPAND’) to target psychosocial determinants of UVR dose to the face in patients with XP.^46^

The unexpectedly large variation in photoprotection behaviour between patients with XP raises the possibility that there may be a similarly dramatic range in behaviour affecting acquired disease-modulating factors between patients with other monogenic disorders, particularly in diseases where the avoidance of a disease-aggravating environmental factor or exposure to a protective environmental factor (such as a therapy) may be a potential source of psychological or social stress. If this is the case, identifying the psychological and social determinants of this variation in behaviour in order to design behaviour change interventions may be a useful approach to improving clinical outcomes in other monogenic disorders.

## Data Availability

All data relevant to the study are included in the article or uploaded as supplementary information. Not applicable.
